# Corticosteroid Injection in the Management of Dupuytren Nodules: A Review of the Literature

**Published:** 2015-09-25

**Authors:** Aditya Sood, Paul J. Therattil, Hee Jin Kim, Edward S. Lee

**Affiliations:** Division of Plastic Surgery, Department of Surgery, Rutgers University–New Jersey Medical School, Newark

**Keywords:** Dupuytren nodules, Dupuytren disease, Dupuytren’s, steroid injection, steroid

Dear Sir,

Dupuytren nodules form early in the course of the disease and usually precede the development of joint contracture. These nodules can present with or without pain and can be multiple in numbers (Fig. 1). Currently, although nodules may be symptomatic with pain, no specific intervention is recommended. Treatment is generally delayed until joint contracture develops.

There are numerous histopathological studies on the composition of Dupuytren nodules, mechanisms of disease progression, and potential cellular interaction with steroids, but there is a lack of clinical trials on the effect of steroid injections into Dupuytren nodules.

Only one study has been completed to determine whether intralesional steroid injections into Dupuytren nodules soften and flatten nodules as well as hasten disease.^1^ In the mentioned study, 63 patients with Dupuytren nodules in early stages of disease underwent a series of triamcinolone acetonide injections over a 4-year period. Each injection contained 60 to 120 mg of triamcinolone acetonide and was administered directly into nodules of patients with contracture of less than 15°. Sixty-two of 63 patients experienced visible regression of disease with softening and flattening of nodules. Overall, the study reported an average of 3.2 injections per nodule and 97% of patients experiencing softening and flattening of nodules. Although immediate regression of nodules was observed in these patients, most of them experienced recurrence of disease. Around half of the patients who received a series of steroid injections had a recurrence of disease within 1 to 3 years of the last injection, which required further injections. Only one patient required surgery due to the development of contracture after 10 injections. Some complications noted in the study include depigmentation and dermal atrophy at the injection site from collagen degradation. These complications, however, resolved completely in the majority of patients within 6 months. In long-term follow-up over 30 years, 2 female patients had late flexor tendon rupture while playing golf, past the initial 4-year study period. The authors recommend strict adherence to the designed protocol and the importance of intralesional triamcinolone acetonide injections into nodules and not beneath them. This study also reports high patient satisfaction with the process of steroid injections. On the basis of this study, steroid injections into Dupuytren nodules may benefit patients by modifying the progression of disease in its early stages.

Another nonsurgical treatment of Dupuytren disease, contracture specifically, is biological intervention. Although the use of corticosteroid injections in Dupuytren nodules has not been adequately studied in clinical settings, intervention involving localized collagenase injections for contracture or late-stage disease has been extensively studied and tested in clinical trials as an alternative to surgical interventions. Collagenase injection has been shown to reduce contracture and improve hand function. Disease recurrence and long-term safety of collagenase injections have yet to be determined, as do the applications to early symptomatic and asymptomatic nodules.^2^^,^^3^

With sparse literature on the use of steroid injections for early Dupuytren disease or nodules, many practitioners rely on anecdotal experiences. We have adopted a simple algorithm for patients presenting with nodules in the early phase of Dupuytren disease (Fig. 2). For painless nodules, observation would be the most appropriate next step to minimize unnecessary complications per the primary dictum of *primum non nocere*. In patients with painful nodules, observation alone would not fulfill the concept of “do no harm,” since the patient will be left in pain without any intervention. In our practice, patients symptomatic with painful nodules are given the option of steroid injections. Additional clinical evidence to support this practice of steroid injections in early phase of Dupuytren disease would be beneficial. In addition, since the differential diagnosis of a palmar mass includes malignancy, biopsy would be indicated if there is a concern for other pathology. Many of these patients initially presenting with nodules go on to develop cords and subsequently joint contracture, at which point we have better evidence to support surgical and nonsurgical interventions addressed previously.

## Figures and Tables

**Figure 1:**
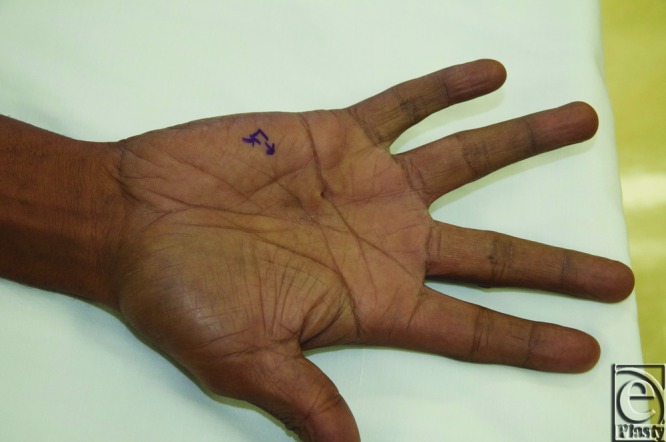
Patient with a painful Dupuytren nodule just proximal to the ring finger distal palmar crease, associated with a pit. This nodule was injected with steroid for its symptomatic presentation.

**Figure 2:**
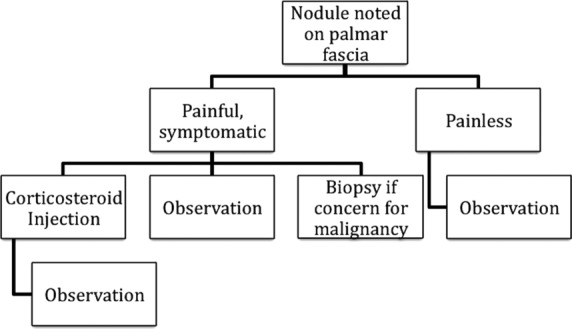
Treatment algorithm for patients presenting with nodules early in the course of Dupuytren disease. Patients with painless nodules are observed, whereas patients with symptomatic or painful nodules are given the option of corticosteroid injections or observation. If nodules are suspicious for malignancy, biopsy is indicated.
